# GENDER DIFFERENCES IN RECEIVING ASSISTANCE WITH DAILY CARE NEEDS AMONG OLDER BLACK AMERICANS LIVING WITH DIABETES

**DOI:** 10.1093/geroni/igz038.1899

**Published:** 2019-11-08

**Authors:** Ledric D Sherman, Chanee D Fabius, Timethia Bonner, Erica Spears

**Affiliations:** 1 Texas A&M University, College Station, Texas, United States; 2 Johns Hopkins University, Baltimore, Maryland, United States; 3 Centers for Disease Control and Prevention, Atlanta, Georgia, United States; 4 Auburn University, Auburn, Alabama, United States

## Abstract

Older Black Americans living with diabetes often receive social support critical to their self-care management practices and quality of life. Studies have reported positive relationships between support and diabetes care among this population, although gender differences exist, with men reporting better quality of life outcomes associated with diabetes self-management than women. More information is needed to to assist healthcare providers indeveloping gender-tailored interventions to improve diabetes self-management. Using data from the 2015 National Health and Aging Trends Study (NHATS; Round 5), a nationally representative sample of Medicare beneficiaries aged 65 and older, our cross-sectional study describes gender differences in receiving assistance with self-care, mobility, and household needs among older Black adults with diabetes (N=621). Participants were majority female (59% Females; 41%Males). Bivariate analyses showed women were often older with fewer years of education, lower incomes, and were more likely to live with others than men. A larger share of respondents reported receiving assistance with household activities (34%; e.g. shopping, medication administration), followed by self-care (21%; e.g. bathing, dressing), and mobility tasks (17%; e.g. getting around inside and outside of the house). Binary logistic regression showed that women were more likely to report receiving assistance with all three tasks after adjusting for age, education, income, living arrangements, number of health conditions, and self-rated health. Future research should identify the relationships between caregivers and care recipients (e.g. spouse/partner, children), as receiving support with daily needs has the potential to impact both the health and quality of life of both caregivers and care-recipients.

